# A Rare *PTF1A* Enhancer Mutation Causing Neonatal Diabetes Mellitus with Pancreatic Agenesis: Case Report and Considerations for Genetic Evaluation

**DOI:** 10.5812/ijem-158056

**Published:** 2025-01-30

**Authors:** Mahdi Paksaz, Hedieh Saneifard, Alimohammad Mirdehghan, Asieh Mosallanejad, Marjan Shakiba, Mohammad Saberi

**Affiliations:** 1 Student Research Center, Faculty of Medicine, Shahid Beheshti University of Medical Sciences, Tehran, Iran; 2 Department of Pediatric Endocrinology and Metabolism, Mofid Children's Hospital, Shahid Beheshti University of Medical Sciences, Tehran, Iran; 3 Shahid Beheshti University of Medical Sciences, Tehran, Iran; 4 Tehran University of Medical Sciences (TUMS), Tehran, Iran

**Keywords:** Neonatal Diabetes Mellitus, *PTF1A* Enhancer Mutation, Pancreatic Agenesis, Permanent Neonatal Diabetes Mellitus

## Abstract

**Introduction:**

Neonatal diabetes mellitus (NDM) is a rare disorder characterized by impaired blood glucose regulation that manifests before six months of age. Unlike autoimmune diabetes, NDM is caused by genetic mutations. One of the rarest causes of NDM is pancreatic agenesis, which results from mutations affecting the pancreas transcription factor 1A (*PTF1A*) gene and its enhancer. The following case report presents a rare instance of this condition.

**Case Presentation:**

This report describes a 2-year-old male child born to consanguineous Iranian parents, diagnosed with NDM due to pancreatic agenesis caused by a rare mutation in the *PTF1A* enhancer. Hyperglycemia was detected from the first day of life, and ultrasonography confirmed the absence of pancreatic tissue. Molecular analysis revealed homozygosity for the g.23508437A > G variant within the enhancer region of the *PTF1A* gene. At two years of age, with pancreatic enzyme replacement and insulin therapy, the patient exhibits normal neurological development, and his physical growth is at the 38th percentile.

**Conclusions:**

Based on previous studies, the g.23508437A > G variant in the *PTF1A* gene enhancer region should be considered in cases of pancreatic agenesis. While whole-exome sequencing (WES) remains the gold standard for genetic diagnosis, it may fail to detect certain mutations. Therefore, targeted evaluation of *PTF1A* is essential when a genetic etiology is suspected.

## 1. Introduction

The onset of diabetes mellitus before the sixth month of life is referred to as neonatal diabetes mellitus (NDM) (1). The estimated prevalence of this condition is approximately 1 in every 90,000 to 160,000 live births (2). Unlike other forms of diabetes, NDM is typically non-autoimmune and is most often caused by monogenic mutations (3). Clinically, NDM is classified into two main types: Transient neonatal diabetes mellitus (TNDM) and permanent neonatal diabetes mellitus (PNDM) (4).

Most cases of TNDM are associated with an imprinted region on chromosome 6q24, which may be affected by paternal uniparental disomy, paternal duplication, or defective methylation of the maternal allele. In some cases, defective methylation is due to biallelic mutations in the *ZFP57* gene, which regulates DNA methylation (5). In contrast, PNDM is predominantly caused by heterozygous mutations in the *KCNJ11* and *ABCC8* genes, which encode subunits of the ATP-sensitive potassium (KATP) channel in pancreatic beta cells. These mutations account for approximately 31% and 10% of PNDM cases, respectively, in Western populations. Patients with *KCNJ11* and *ABCC8* mutations typically present with isolated diabetes, and many can transition from insulin therapy to oral sulfonylureas, providing an effective alternative treatment strategy (6).

Complete pancreatic agenesis has been documented in only a small number of cases and exhibits genetic heterogeneity (7). Several genes, including *PDX1*, *PTF1A*, *HNF1B*, and *GATA6*, have been implicated in pancreatic agenesis (4). Exocrine pancreatic insufficiency (EPI) in infants is often identified through symptoms such as failure to thrive, chronic diarrhea, anemia, and hypoalbuminemia, with fecal elastase-1 being the most commonly used diagnostic test. Pancreatic agenesis, whether isolated or syndromic, can result in both exocrine and endocrine pancreatic insufficiency, leading to significant developmental complications (8). An international cohort study of neonates diagnosed with diabetes mellitus within the first six months of life reported that pancreatic agenesis was present in only 4.9% of cases (9).

The *PTF1A* (pancreas-specific transcription factor 1A) gene plays a critical role in pancreatic and cerebellar development by encoding pancreas transcription factor-1-alpha, a basic helix-loop-helix protein (10). Mutations in a recently identified distal developmental enhancer of *PTF1A* have been linked to pancreatic agenesis in 14 individuals, including 10 probands and 4 family members. These cases of pancreatic agenesis were not associated with cerebellar involvement or other extra-pancreatic symptoms (11). While the study by Weedon et al. (11) initially reported 14 cases of pancreatic agenesis due to mutations in the distal *PTF1A* enhancer, more recent research by Demirbilek et al. expanded the cohort to 30 individuals, including cases of diabetes associated with *PTF1A* enhancer mutations (12).

Building on previous reports, the current study identifies a rare mutation linked to pancreatic agenesis and NDM.

## 2. Case Presentation

The male neonate, born to consanguineous Iranian parents, was brought in at 33 days old with persistent hyperglycemia, initially recorded at a previous medical center. The pregnancy was complicated by oligohydramnios. He was delivered via cesarean section at 35 weeks of gestation due to an abnormal non-stress test suggesting possible fetal distress. At birth, his body measurements were as follows: Length of 41 cm, birth weight of 1,600 g, and head circumference of 31 cm.

A physical examination at this center revealed normal findings, with no skeletal deformities or facial dysmorphism. Hyperglycemia was noted from the first day of life, with recorded blood sugar levels of 181, 700, 900, 400, and 216 mg/dL on different days. All glucose measurements were taken postprandially to minimize the potential risk of an NPO diet affecting glucose levels in neonates.

Laboratory values obtained on the first day of admission included venous blood gas (VBG) results showing a pH of 7.482, pCO_2_ of 26.6 mmHg, pO_2_ of 115 mmHg, and HCO_3_- of 19 mEq/L, indicating stable acid-base status and ruling out diabetic ketoacidosis (DKA). The blood glucose level on admission was 250 mg/dL, prompting initiation of NPH insulin therapy.

Laboratory tests suggested fat malabsorption, with stool analysis revealing an increased excretion of neutral fat (more than 100 drops) and loose consistency. Fecal elastase levels were below 21 mg/g (normal range: 200 - 500 mg/g). The hemoglobin level was 9.2 g/dL, with an MCV of 91.6 fL and an RDW of 14.8%, indicating anemia. Additionally, total protein was recorded at 3.5 g/dL (normal newborn range: 4.1 - 6.31 g/dL), and amylase was measured at 78 U/L, supporting the diagnosis of exocrine pancreatic insufficiency. Ophthalmoscopic examination, as well as thyroid and renal function tests, yielded normal results. Other laboratory findings were within normal limits.

An abdominal ultrasound was performed, but no pancreatic tissue could be identified. The absence of pancreatic tissue was confirmed through additional imaging studies, including an abdominal MRI without contrast. No other abnormalities were detected. Brain imaging, including both ultrasound and MRI, was entirely normal.

The patient was started on NPH insulin at a dose of 0.3 IU twice daily and exogenous pancreatic enzyme replacement therapy with Creon.

**Figure 1.**
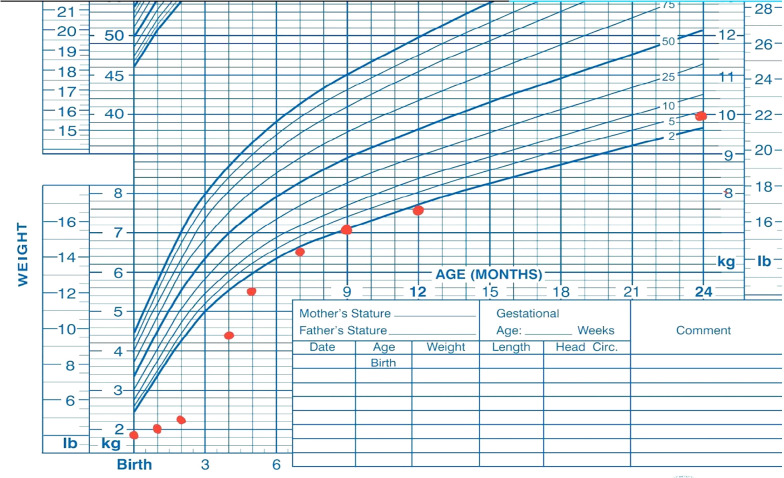
Growth chart of the infant (both weight and height growth)

At the latest follow-up visit, at 2 years and 7 months of age, the patient weighed 10 kg (38th percentile) (Figure 1) and exhibited normal development with a typical neurological and general physical examination. His growth velocity was recorded at 9 cm per year. Routine follow-up visits are conducted every three months, involving both a gastroenterologist and an endocrinologist to monitor malabsorption, growth, development, and HbA1c levels.

Regarding blood glucose management, the patient's HbA1c levels over the past five visits ranged from 7.9% to 9.6%, with specific values of 9.0%, 9.6%, 9.3%, 9.1%, and 7.9%. This represents a significant improvement from initial HbA1c levels of 13.5% and 11%. Continuous monitoring and treatment adjustments are ongoing to optimize glucose control and support long-term management goals.

Screening for potential microvascular and macrovascular complications related to diabetes will be initiated at an appropriate age, following standard guidelines for early detection and management.

The patient remains on exogenous pancreatic enzyme replacement therapy with Creon, and his insulin regimen consists of NPH at 1.5 IU before each meal (three times daily).

## 3. Gene Analysis

During genetic and molecular investigations, whole-exome sequencing (WES) was performed, but no pathogenic mutations were identified. Given the patient's pancreatic agenesis, a targeted analysis of potential mutations in the enhancer region of the *PTF1A* gene — previously documented in OMIM — was pursued as a secondary diagnostic approach.

PCR amplification followed by Sanger sequencing was conducted to detect variants in a specific ~450 bp enhancer region, located within intron 15 of the *C10orf67* gene. The *PTF1A* and *C10orf67* genes are both located on chromosome 10. Although separated by approximately 1.5 Mb, potential regulatory interactions, such as shared enhancers, have been suggested.

**Figure 2.**
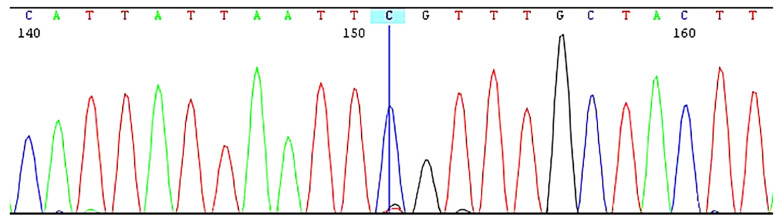
Sequence data of the amplified *PTF1A* gene enhancer (*C10orf67* intronic) region. The sequence revealed c.1570 + 4090T > C mutation in the homozygous state.

Sequence analysis of the *PTF1A* enhancer region revealed the presence of a c.1570 + 4090 T > C (g.23508437A > G) mutation in a homozygous state (Figure 2). This mutation is located approximately 50 kb from the *PTF1A* gene. The spatial proximity is notable, as regulatory elements like enhancers can influence the expression of nearby genes. *PTF1A* plays a pivotal role in pancreatic development, and alterations in its expression could disrupt normal pancreatic organogenesis. However, further experimental studies are needed to confirm the precise molecular mechanism underlying this regulatory interaction.

## 4. Discussion

A rare form of diabetes that manifests as hyperglycemia within the first six months of life is known as NDM. The most commonly observed clinical findings include intrauterine growth retardation (IUGR), failure to thrive, and reduced C-peptide levels (6). From a clinical standpoint, NDM is divided into two primary subtypes: TNDM and PNDM. The TNDM is characterized as a developmental disorder in insulin production that generally resolves spontaneously as the child grows, whereas PNDM persists without remission (4). The leading causes of PNDM include heterozygous activating mutations in the *KCNJ11* and *ABCC8* genes, which result in inhibited insulin secretion for glucose regulation, although pancreatic development remains unaffected in these cases (8). One of the rare etiologies of PNDM is pancreatic agenesis, which is caused by heterogeneous mutations (3). While coding mutations in genes such as *PTF1A*, *GATA6*, and *GATA4* directly alter protein function, mutations in non-coding regions, such as enhancers, affect the regulation of key developmental genes, including *PDX1*, *MNX1*, and *NKX6-1*. Miguel-Escalada et al. demonstrated that mutations in the *PTF1A* enhancer disrupt a regulatory network critical for pancreas development, leading to organ agenesis (13).

The *PTF1A* gene plays a crucial role in encoding pancreas transcription factor-1 alpha, which is essential for the development of both the pancreas and cerebellum (10). The specific site of *PTF1A* mutations, whether coding or non-coding, influences the phenotypic traits of affected individuals. This further underscores the vital role of non-coding sequences in the endocrine and exocrine development of the pancreas (6). The *PTF1A* enhancer is particularly important in endocrine pancreatic development, initiating a cascade of epigenetic priming events in multipotent progenitor cells (MPCs) and their descendants (13). The Chr10.23508437A > G variant has been identified as the most frequently occurring mutation in the *PTF1A* enhancer (5).

Here, we report a 2-year-old patient diagnosed with PNDM due to pancreatic agenesis caused by a novel mutation in the *PTF1A* enhancer. The patient, initially 33 days old, presented to the Emergency Department with persistent hyperglycemia. Neonatal hyperglycemia can result from various etiologies, including transient hyperglycemia due to stressors such as infection, hypoxia, or asphyxia, as well as medication-induced hyperglycemia, commonly caused by corticosteroids or inotropes. A thorough clinical history, including a review of any recent medications and stressors, is crucial to ruling out these common causes. In this case, no relevant medical history or medication use was identified that could explain the hyperglycemia. Additionally, cultures from blood, urine, and cerebrospinal fluid (CSF) were all negative, ruling out infectious causes (14).

Exocrine pancreatic insufficiency in infants is often identified through symptoms such as failure to thrive, chronic diarrhea, anemia, and hypoalbuminemia, with fecal elastase-1 being the most commonly used diagnostic test. Pancreatic agenesis, whether isolated or syndromic, can lead to both exocrine and endocrine pancreatic insufficiency, resulting in significant developmental complications (8). To biochemically confirm EPI, fecal elastase and pancreatic enzyme levels (pancreatic amylase and lipase) were measured and assessed according to laboratory-specific reference values [11]. Laboratory findings for this patient indicated fat malabsorption, with stool analysis showing over 100 droplets of neutral fat and reduced fecal elastase (< 21 mg/g; normal: 200 - 500 mg/g). Hemoglobin was 9.2 g/dL, MCV 91.6 fL, and RDW 14.8%, suggesting anemia. Total protein was 3.5 g/dL (normal: 4.1 - 6.31 g/dL), and amylase was 78 U/L, further supporting the diagnosis of exocrine pancreatic insufficiency.

Both ultrasound and MRI were used to confirm the absence of the pancreas, highlighting their complementary strengths in diagnosis. Ultrasound, often the first-choice imaging modality due to its non-invasive nature and rapid results, effectively identifies major anatomical abnormalities but may lack the resolution to detect fine pancreatic structures. To address this limitation, MRI was performed, offering superior soft-tissue contrast and a detailed assessment of pancreatic structures and adjacent tissues. This combined imaging approach follows best clinical practices, ensuring a comprehensive and accurate diagnosis of pancreatic agenesis (15).

Genetic and molecular investigations included WES, which did not reveal any pathogenic mutations. Given the patient's pancreatic agenesis, a targeted analysis of the *PTF1A* enhancer region was performed using PCR amplification and Sanger sequencing of a ~450 bp region within intron 15 of the *C10orf67* gene. This analysis confirmed homozygosity for the c.1570 + 4090 T > C variant.

De Franco and Ellard highlighted the advantages of whole-genome sequencing (WGS), WES, and targeted next-generation sequencing in diagnosing neonatal diabetes. While WES is cost-effective and focuses on coding regions, it is limited in detecting mutations in non-coding regions, such as regulatory elements crucial for conditions like pancreatic agenesis. WGS, on the other hand, provides a comprehensive genetic analysis, identifying both coding and non-coding mutations, including structural variations that are often missed by WES (3). Targeted sequencing, when a specific genetic cause is suspected, offers a focused and cost-effective diagnostic approach. The authors emphasize that WGS is superior for detecting non-coding mutations and structural variations, making it a more thorough diagnostic tool compared to WES.

In this case of pancreatic agenesis, WES failed to identify pathogenic mutations, likely due to its inability to assess non-coding regions. As a result, a targeted analysis of the *PTF1A* enhancer region was conducted, confirming the presence of the g.23508437 A > G variant. While Sanger sequencing is effective for detecting small variants, it cannot identify large deletions or duplications, highlighting its complementary role alongside WES.

Given the diagnostic challenges encountered, we suggest that evaluating enhancer regions of genes like *PTF1A* is crucial in cases of NDM with gastrointestinal involvement, especially when WES results are negative. Non-coding mutations, which WES often fails to detect, can be pivotal in complex developmental disorders. Targeted sequencing provides a more accurate and focused approach to identifying these mutations, enhancing diagnostic precision and improving our understanding of disease mechanisms (15). This underscores the importance of investigating non-coding regions for pathogenic mutations that might otherwise go undetected.

The c.1570 + 4090 T > C mutation in the *PTF1A* enhancer region is believed to impair regulatory elements essential for pancreatic development, potentially causing pancreatic agenesis. This mutation, along with others in the enhancer region, has been linked to diabetes and pancreatic abnormalities (14).

The clinical features and genetic analysis of 29 previously published case reports, organized chronologically in Table 1, further emphasize that the c.1570 + 4090T > C mutation in the *PTF1A* enhancer region is strongly associated with pancreatic agenesis. This review consolidates key findings, reinforcing the mutation’s significant role in the pathogenesis of pancreatic agenesis.

**Table 1.** Clinical Reports of Pancreatic Agenesis Sort by Publication Date

No.	Ref. No.	Gestat. Age (wk)	Birth Weight (g)	Presentation Age	Country	Pub. Date	Mutation
**1**	(16)	39	2800	4 weeks	-	2008	-
**2**	(16)	39	2400	3 weeks	-	2008	-
**3**	(16)	38	3000	78 weeks	-	2008	-
**4**	(16)	38	2300	2 weeks	-	2008	-
**5**	(17)	39	1660	2 days	Turkey	2009	Not checked
**6**	(18)	36	< third precentile	2 months	Saudi Arabia	2011	c.437-460del
**7**	(19)	35	1450	10 days	turkey	2015	Homozygous for g.23508437 A > G
**8**	(19)	38	2600	9 years	Turkey	2015	Homozygous for g.23508437 A > G
**9**	(5)	32	1200	3 weeks	Turkey	2015	Homozygous for g.23508365 A > G
**10**	(5)	39	2400	10 weeks	Turkey	2015	Homozygous for g.23508437 A > G
**11**	(5)	31	1500	1 week	Turkey	2015	Homozygous for g.23508437 A > G
**12**	(7)	38	1980	1 day	Saudi Arabia	2016	c.571C > A
**13**	(7)	37	2000	1 day	Saudi Arabia	2016	c.571C > A
**14**	(7)	34	1275	1 day	Kuwait	2016	-
**15**	(7)	36	1400	8 days	Kuwait	2016	-
**16**	(9)	37	1935	7 days	European	2017	Compound hetero g.23508442 A > G
**17**	(20)	38	1100	1 day	European	2017	-
**18**	(4)	37	1900	1 month	Turkish	2018	Homozygous g.23508363 A > G/*PTF1A*
**19**	(4)	37	1520	44 days	Turkish	2018	Homozygous g.23508437 A > G/*PTF1A*
**20**	(6)	Term	1300	1 day	Qatar	2019	*PTF1A* (chromosome 10:23502416–23510031)
**21**	(6)	Term	1000	1 day	Qatar	2019	*PTF1A* (chromosome 10:23502416–23510031)
**22**	(6)	Term	1900	1 day	Qatar	2019	*PTF1A* (chromosome 10:23502416–23510031)
**23**	(1)	39	1800	17 days	Iranian	2021	g.23508441 T > G
**24**	(2)	-	2600	6 days	Saudi Arabia	2023	*PTF1A* homozygous mutation (c.5171C > A p.(Pro191Thr).
**25**	(2)	Full term	1600	1 day	Saudi Arabia	2023	*PTF1A* homozygous mutation (c.5171C > A p.(Pro191Thr)
**26**	(2)	Full term	2000	4 day	Saudi Arabia	2023	*PTF1A* homozygous mutation (c.5171C > A p.(Pro191Thr).
**27**	(2)	Term	2600	2 days	Saudi Arabia	2023	*PTF1A* homozygous mutation (c.5171C > A p. (Pro191Thr).
**28**	(2)	Term	1300	2 days	Saudi Arabia	2023	*PTF1A* homozygous mutation c.5171C > A p.(Pro191Thr)
**29**	(2)	Term	2200	12 days	Saudi Arabia	2023	A *PTF1A* homozygous mutation c.5171C > A p.(Pro191Thr).
**30**	This case	35	1600	33 days	Iran	2024	Homozygosity for the g.23508437A > G

Abbreviations: Ref.No., reference number; Gestat. Age, gestational age; wk, week (s); g, gram (s); Pub. Date, publication date.

### 4.1. Management and Prognosis

In this report, NPH insulin was selected as the initial treatment for diabetes resulting from a *PTF1A* enhancer mutation, following established neonatal diabetes protocols. As an intermediate-acting insulin, NPH balances basal and prandial insulin coverage, making it suitable for managing insulin deficiency due to pancreatic agenesis. Its flexibility in dosing simplifies blood glucose management throughout the day. Adjustments were made based on frequent glucose monitoring, aligning with standard neonatal diabetes management protocols (14). This individualized approach, based on guidelines from existing literature, aimed to maintain optimal glycemic control while minimizing the risk of hypoglycemia and hyperglycemia. Regular follow-up allowed for continuous insulin adjustments as the patient's needs evolved, particularly given the long-term nature of diabetes associated with *PTF1A* mutations.

Managing blood glucose in infants presents significant challenges due to the high risk of hypoglycemia. Short-acting insulins are limited by their unpredictable effects and the potential to cause dangerous blood sugar drops. NPH insulin, despite its complexity, was chosen to reduce this risk. Parental education on proper insulin dilution and administration was crucial, necessitating ongoing training and support. However, hypoglycemia risk remains, requiring continuous monitoring.

The prognosis for patients with pancreatic agenesis is influenced by genetic, developmental, and clinical factors, with early diagnosis and comprehensive management playing key roles in improving survival and quality of life. As mentioned, careful monitoring of growth and development has been conducted, and continued follow-up visits are expected to support a more favorable prognosis (14).

## Data Availability

The dataset presented in the study is available on request from the corresponding author during submission or after publication. The data are not publicly available due to the patient's privacy policies of the Mofid Childrens Hospital.
